# Determinants of severity among hospitalised COVID-19 patients: Hospital-based case-control study, India, 2020

**DOI:** 10.1371/journal.pone.0261529

**Published:** 2021-12-29

**Authors:** Sanjay P. Zodpey, Himanshu Negandhi, Vineet Kumar Kamal, Tarun Bhatnagar, Parasuraman Ganeshkumar, Arvind Athavale, Amiruddin Kadri, Amit Patel, A. Bhagyalaxmi, Deepak Khismatrao, E. Theranirajan, Getrude Banumathi, Krishna Singh, P. Parameshwari, Prasita Kshirsagar, Rita Saxena, Sanjay G. Deshpande, Kadloor Satyanand, Saurabh Hadke, Simmi Dube, Sudarshini Subramaniam, Surabhi Madan, Swapnali Kadam, Tanu Anand, Kathiresan Jeyashree, Manickam Ponnaiah, Manish Rana, Manoj V. Murhekar, DCS Reddy

**Affiliations:** 1 Public Health Foundation of India, Gurgaon, Haryana, India; 2 ICMR National Institute of Epidemiology, Chennai, Tamil Nadu, India; 3 Chirayu Medical College & Hospital, Bhopal, Madhya Pradesh, India; 4 B.J. Medical College, New Civil Hospital, Ahmedabad, Gujarat, India; 5 Care Institute of Medical Sciences, Ahmedabad, Gujarat, India; 6 Symbiosis Hospital and Research Centre, Pune, Maharashtra, India; 7 Institute of Community Medicine, Madras Medical College, Chennai, Tamil Nadu, India; 8 Chengalpattu Medical College, Chengalpattu, Tamil Nadu, India; 9 Rajiv Gandhi Medical College, Chhatrapati Shivaji Maharaj Hospital, Maharashtra, Thane, India; 10 Gandhi Medical College, Bhopal, Madhya Pradesh, India; 11 Datta Meghe Medical College, Meghe Hospital and Research Centre, Nagpur, Maharashtra, India; 12 Indian Council of Medical Research (ICMR), New Delhi, India; 13 GMERS Medical college, Ahmedabad, Gujarat, India; 14 Independent consultant, Lucknow, Uttar Pradesh, India; University Magna Graecia of Catanzaro, ITALY

## Abstract

**Background:**

Risk factors for the development of severe COVID-19 disease and death have been widely reported across several studies. Knowledge about the determinants of severe disease and mortality in the Indian context can guide early clinical management.

**Methods:**

We conducted a hospital-based case control study across nine sites in India to identify the determinants of severe and critical COVID-19 disease.

**Findings:**

We identified age above 60 years, duration before admission >5 days, chronic kidney disease, leucocytosis, prothrombin time > 14 sec, serum ferritin >250 ng/mL, d-dimer >0.5 ng/mL, pro-calcitonin >0.15 μg/L, fibrin degradation products >5 μg/mL, C-reactive protein >5 mg/L, lactate dehydrogenase >150 U/L, interleukin-6 >25 pg/mL, NLR ≥3, and deranged liver function, renal function and serum electrolytes as significant factors associated with severe COVID-19 disease.

**Interpretation:**

We have identified a set of parameters that can help in characterising severe COVID-19 cases in India. These parameters are part of routinely available investigations within Indian hospital settings, both public and private. Study findings have the potential to inform clinical management protocols and identify patients at high risk of severe outcomes at an early stage.

## Introduction

COVID-19 pandemic has caused over 2.4 million deaths and over 111 million cases worldwide by 24^th^ February 2021 [1]. Due to widespread transmission, several countries were burdened with high case load and deaths. Critical care resources have been stretched across some countries [2, 3]. The fatalities reported by countries and regions also varied widely.

While the available data on absolute number of deaths is fairly reliable, the calculation of mortality rates and comparing them across countries is difficult because countries widely differ in their screening and testing criteria. The analysis of 72,314 cases using data from the Chinese Centre for Disease Control and Prevention [4], indicated most cases to be mild (81%; i.e., non-pneumonia and mild pneumonia), whereas 14% were severe (i.e., dyspnea, respiratory frequency ≥30/min, blood oxygen saturation ≤93%, the partial pressure of arterial oxygen to fraction of inspired oxygen ratio <300, and/or lung infiltrates >50% within 24 to 48 hours), and 5% were critical (i.e., respiratory failure, septic shock, and/or multiple organ dysfunction or failure). India rapidly scaled up hospital and critical care resources and a proactive public health response targeting surveillance, wearing masks, limiting movement in the early phase of the epidemic along with an intensive information dissemination campaign. The mortality attributed to COVID-19 in India was relatively low compared to the rest of the world. India had reported 1,19,71,624 cases and 1,61,552 deaths till 28 March 2021 with lowest case fatality ratio of 1.5% globally [5].

The determinants of severity can guide clinical management; proactively screening for their presence could prioritize COVID-19 patients for intensive care treatment and thereby allocate scarce medical resources appropriately. Risk factors for the development of severe disease and death have been widely reported across several studies [6], and vulnerable groups include older adults, cardiovascular disease, diabetes, chronic respiratory disease, hypertension, and cancer. Obesity and smoking were also associated with increased risks in some studies [6]. Lymphopenia is a predictor of disease progression [7]. Cytokine storm is also associated with disease severity [8].

Knowledge of characteristics of people at high risk of experiencing a poor outcome from the infection could help in care provision [9]. We conducted this study to identify the determinants of severe COVID-19 disease in India using a case-control study design.

## Methods

### Study design and setting

We did a hospital-based case-control study among laboratory-confirmed COVID-19 patients of age≥18 years, newly admitted to nine designated COVID-19 hospitals (both public and private), from six cities across India during September—November 2020. The study centres included BJ Medical College, Ahmedabad; Care Institute of Medical Sciences (CIMS Hospital), Ahmedabad; Gandhi Medical College, Bhopal; Chirayu Medical College & Hospital, Bhopal; Symbiosis Hospital and Research Centre, Pune; Rajiv Gandhi Medical College and CSMH, Kalwa, Thane; Madras Medical College, Chennai; Chengalpattu Medical College, Chengalpattu; and Datta Meghe Medical College, Wanadongari, Nagpur.

Cases (severe disease, at admission) and controls (mild disease at admission) were defined as per the Government of India’s COVID-19 case management guidelines (version 5; issued on 03/07/2020) (S1 Table) [10]. The working definition of severe COVID-19 disease included death and/or development of severe disease requiring ICU admission and/or ventilator support.

### Sample size

Assuming an exposure rate of risk factors as 9% among controls (prevalence of hypertension in India) [11], anticipated Odds Ratio (OR) of 2.3 [12], at 5% level of significance and 90% power, we estimated a sample size of 244 cases and controls, each.

### Selection of study participants

Cases and controls were identified from the admission records of study hospitals and those found to fulfil the eligibility criteria were selected consecutively until the desired sample size was achieved.

### Data collection

We did face-to face interviews with the patients using a structured questionnaire to collect data on socio-demographic details, concurrent disease conditions and clinical symptomatology. For concurrent disease conditions questions were included about duration, severity and medication. If the patient was unable to respond, the close family members of the patient were interviewed. Data pertaining to clinical and laboratory variables were extracted from the hospital records using a data abstraction form. All information pertained to the duration between development of symptoms and the time of admission of the patients in the study hospitals.

### Data analysis

Categorical and continuous variables were represented as frequency (percentage) and median (Interquartile range (IQR)), respectively. Between cases and controls, categorical variables were compared using Chi-square/Fisher’s exact test, whichever applicable. Non-normally distributed continuous variables (examined using Shapiro-Wilk test) were compared using the Wilcoxon rank-sum test. The quantification of association was represented as crude and adjusted odds ratios with 95% confidence intervals (CI) using simple and multiple logistic regression analysis, respectively. Factors with p-value <0.25 in simple logistic regression analysis and/or clinical relevance, with the exclusion of those operating through a common clinical pathway or indicating similar pathology, were selected for the inclusion in the final model based on multiple logistic regression analysis, after checking for collinearity using variance inflation factor (VIF). Each factor was adjusted for relevant and measured confounders identified using directed acyclic graphs and -2 log likelihood ratio test. Data analysis was done using Stata V.15.1 software.

### Ethical issues

Written informed consent was obtained from study participants. The study protocol was approved by the Institutional Ethics Committee of the Indian Institute of Public Health—Delhi. The protocol was also approved by the institutional ethics committees of all study sites.

## Results

We included 244 patients with severe COVID-19 disease (Cases) and 245 with mild to moderate COVID-19 disease (Controls). Compared to the controls, a significantly higher proportion of cases were more than 60 years old, had lower monthly household income, less educated, and possessed a below poverty line (BPL) card. (Table 1).

**Table 1:** Background characteristics of cases and controls with COVID-19 in India, 2020.

Characteristics	Cases			Controls			p-value
	N	n/ Median	%/IQR	N	n/ Median	%/IQR	
Age (years)	244	58.9	(48.1–66.6)	245	45.7	(31.9–56.0)	
18–45		45	18.5		118	48.2	<0.001
46–60		85	34.8		87	35.5	
>60		114	46.7		40	16.3	
Gender	244			245			
Male		165	67.6		175	71.4	0.361
Female		79	32.4		70	28.6	
Body mass index (BMI) (kg/m^2^)	242	25.7	(23.0–29.3)	245	25.5	(23.0–28.1)	
≤27.5		156	64.5		172	70.2	0.177
>27.5		86	35.5		73	29.8	
Average monthly household income (INR)	223	20,000	(10,000–40,000)	238	25,000	(10,000–50,000)	0.007
Years of education	244	10	(5–12)	245	12	(10–15) *	<0.001
Possess BPL card	244	79	32.4	245	43	17.5	<0.001
Migrant	242	28	11.6	245	35	14.3	0.372
Current smoker	241	40	16.6	244	41	16.8	0.952
H/o BCG vaccination	236	158	66.9	242	196	80.9	<0.001

IQR–inter quartile range

The most common symptoms at admission were fever, shortness of breath, cough and myalgia. A significantly higher proportion of cases reported cough and presented with hypertension, diabetes mellitus and chronic kidney disease. Cases also had a significantly higher proportion of multiple comorbidities compared to the controls. (Table 2).

**Table 2:** Clinical characteristics of cases and controls at the time of admission in India, 2020.

Characteristics	Cases (n = 244)		Controls (n = 245)		p-value
	n	%	n	%	
**Presenting symptoms**					
Temperature > 37.8 °C (100 °F)	201	82.4	198	80.8	0.656
Cough	169	69.2	128	52.2	<0.001
Myalgia/ pain & aches in the body	93	38.1	103	42.0	0.376
Sore throat	50	20.5	63	25.7	0.171
Headache	24	9.8	57	23.7	<0.001
Diarrhoea	13	5.3	26	10.6	0.031
Runny nose	15	6.1	21	8.6	0.305
Vomiting	15	6.1	15	6.1	0.991
Seizures	6	2.4	1	0.4	0.056
**Co-morbidities**					
Hypertension	119	48.7	58	23.7	<0.001
Diabetes mellitus	99	40.6	48	19.6	<0.001
Chronic Kidney Disease	13	5.3	1	0.4	<0.001
Cardiovascular disease	8	3.3	4	1.6	0.239
Asthma	4	1.6	4	1.6	0.995
Chronic lung disease	3	1.2	3	1.2	0.996
Chronic Heart Disease	4	1.6	2	0.8	0.408
Others	33	13.5	37	15.1	0.618
**Number of comorbidities**					
None	79	32.4	137	55.9	<0.001
Single	70	28.7	64	26.1	
Multiple	95	38.9	44	17.9	

A significantly higher proportion of cases compared to controls had abnormal laboratory parameters at the time of admission, except for blood group, creatinine kinase and vitamin D. (Table 3).

**Table 3:** Laboratory parameters of cases and controls with COVID-19 at the time of admission in India, 2020.

Characteristics	Cases			Controls			p-value
	N	n	%	N	n	%	
**Blood group**	239			245			
A		53	22.2		56	22.9	0.795
B		85	35.6		21	8.6	
AB		25	10.5		82	33.5	
O		76	31.8		86	35.1	
**Abnormal parameters**							
Leucocytosis (TWBC>11000 /mm^3^)	244	115	47.1	245	35	14.3	<0.001
Erythrocyte Sedimentation Rate >30 mm/hr	244	129	52.9	245	95	38.78	0.002
Prothrombin time > 14 sec	244	146	59.8	244	100	40.9	<0.001
Activated partial thromboplastin time >40 sec	244	51	20.9	244	37	15.16	0.099
Serum ferritin >250 ng/mL	244	195	79.9	245	83	33.9	<0.001
D-dimer >0.5 ng/mL	244	171	70.1	245	83	33.9	<0.001
Pro-calcitonin >0.15 μg/L	244	89	36.5	245	17	6.9	<0.001
Fibrin degradation product >5 μg/mL	237	167	70.5	233	105	45.0	<0.001
Serum triglyceride >150 mg/dL	244	109	44.7	245	84	34.3	0.019
C-reactive protein >5 mg/L	244	219	89.7	245	126	51.4	<0.001
Lactate dehydrogenase >150 U/L	243	238	97.9	245	229	93.5	0.015
Creatinine kinase >200 U/L	244	52	21.3	237	47	19.8	0.688
Interleukin-6 >25 pg/mL	244	142	58.2	245	79	32.2	<0.001
Fasting blood sugar >125 mg/dL	244	131	53.7	245	70	28.6	<0.001
Serum homocysteine >15 mcmol/L	244	77	31.6	238	131	55.0	<0.001
Serum calcium < = 8.5 mg/dL	243	120	49.4	237	62	26.2	<0.001
Vitamin D < = 5 ng/mL	244	7	2.9	239	3	1.3	0.213
Neutrophil Lymphocyte Ratio ≥3	239	206	86.2	238	97	40.8	<0.001
Deranged Liver Function Test	244	117	52.0	245	51	20.8	<0.001
Deranged Renal Function Test	244	93	38.1	245	23	9.4	<0.001
Deranged Serum electrolytes	244	103	42.2	245	38	15.5	<0.001

TWBC: Total White Blood Cell Count.

On univariate analysis, age of 60 years and above, duration before admission more than five days, diabetes mellitus, hypertension, chronic kidney disease, leucocytosis, elevated levels of erythrocyte sedimentation rate, prothrombin time, serum ferritin, d-dimer, pro-calcitonin, fibrin degradation products, c-reactive protein, lactate dehydrogenase, interleukin-6, neutrophil lymphocyte ratio (NLR) and deranged liver function tests, renal function tests and serum electrolytes were associated with severe COVID-19 disease. After adjusting for known confounders, factors associated with severe COVID-19 were age above 60 years, duration before admission >5 days, pre-existing diabetes, chronic kidney disease, leucocytosis, prothrombin time > 14 sec, serum ferritin >250 ng/mL, d-dimer >0.5 ng/mL, pro-calcitonin >0.15 μg/L, fibrin degradation products >5 μg/mL, C-reactive protein >5 mg/L, lactate dehydrogenase >150 U/L, interleukin-6 >25 pg/mL, NLR ≥3, and deranged liver function, renal function and serum electrolytes. (Table 4).

**Table 4:** Factors associated with severity among hospitalised COVID-19 patients, India, 2020.

Factors	Unadjusted OR (95% CI)	p-value	Adjusted OR (95% CI)
Age > = 60 years	4.5 (2.9–6.8)	<0.001	**-**
Male gender	0.8 (0.6–1.2)	0.361	-
BMI >27.5	1.3 (0.9–1.9)	0.177	1.1 (0.7–1.8) ^a^
Duration before admission >5 days	1.5 (1.1–2.2)	0.027	1.5 (1.0–2.2) ^b^
Asthma	1.0 (0.2–4.0)	0.995	-
Chronic lung disease	1.0 (0.2–5.0)	0.996	-
Diabetes mellitus	2.8 (1.9–4.2)	<0.001	1.8 (1.1–2.9) ^c^
Chronic Heart Disease	2.0 (0.4–11.1)	0.418	-
Cardiovascular disease	2.0 (0.6–6.9)	0.249	-
Hypertension	3.1 (2.1–4.5)	<0.001	1.5 (0.9–2.3) ^e^
Chronic Kidney Disease	13.7 (1.8–105.8)	0.012	8.7 (1.1–71.6) ^d^
Leucocytosis	5.3 (3.5–8.3)	<0.001	5.2 (3.3–8.2) ^f^
Erythrocyte Sedimentation Rate >30 mm/hr	1.8 (1.2–2.5)	0.002	1.4 (0.9–2.0) ^g^
Prothrombin time > 14 sec	2.1 (1.5–3.1)	<0.001	1.9 (1.3–2.9) ^h^
Activated partial thromboplastin time >40 sec	1.5 (0.9–2.3)	0.100	1.2 (0.7–2.0) ^h^
Serum ferritin >250 ng/mL	7.8 (5.1–11.7)	<0.001	6.2 (4.0–9.7) ^i^
D-dimer >0.5 ng/mL	4.6 (3.1–6.7)	<0.001	3.8 (2.6–5.6) ^j^
Pro-calcitonin >0.15 μg/L	7.7 (4.1–13.4)	<0.001	5.5 (3.1–9.9) ^k^
Fibrin degradation products >5 μg/mL	2.9 (1.9–4.2)	<0.001	3.1 (2.1–4.6) ^l^
C-reactive protein >5 mg/L	8.3 (5.1–13.4)	<0.001	6.7 (4.0–11.1) ^m^
Lactate dehydrogenase >150 U/L	3.3 (1.2–9.2)	0.021	4.6 (1.5–14.2) ^n^
Creatinine kinase >200 U/L	1.1 (0.7–1.7)	0.688	-
Interleukin-6 >25 pg/mL	2.9 (2.0–4.2)	<0.001	2.4 (1.6–3.8) ^o^
Neutrophil lymphocyte ratio ≥3	9.1 (5.8–14.2)	<0.001	5.2 (3.1–8.9) ^p^
Deranged Liver Function Test	3.5 (2.3–5.2)	<0.001	2.8 (1.8–4.3) ^q^
Deranged Renal Function Test	5.9 (3.6–9.8)	<0.001	3.8 (2.2–6.4) ^r^
Deranged Serum electrolytes	4.0 (2.6–6.1)	<0.001	2.3 (1.4–3.7) ^s^

Adjusted for:

^a^—age, diabetes, hypertension, chronic kidney disease, liver function test, renal function test;

^b^—age, diabetes, hypertension, chronic kidney disease;

^c^—age, hypertension, chronic kidney disease, liver function test, renal function test;

^d^—age, diabetes, chronic kidney disease;

^e^—age, diabetes, chronic kidney disease, renal function test;

^f^—diabetes, chronic kidney disease;

^g^—diabetes, chronic kidney disease, renal function test;

^h^—liver function test, renal function test;

^i^—serum electrolytes, liver function test, renal function test;

^j^—liver function test, prothrombin time;

^k^—leucocytosis, erythrocyte sedimentation rate;

^l^—chronic kidney disease, prothrombin time;

^m^—leucocytosis, erythrocyte sedimentation rate;

^n^—liver function test, leucocytosis, erythrocyte sedimentation rate, C-reactive protein;

^o^—leucocytosis, erythrocyte sedimentation rate, C-reactive protein;

^p^—age, body mass index, leucocytosis, erythrocyte sedimentation rate, C-reactive protein, lactate dehydrogenase;

^q^—age, diabetes, hypertension, chronic kidney disease, renal function test;

^r^—age, diabetes, hypertension;

^s^—chronic kidney disease, liver function test, renal function test

## Discussion

We identified older age, co-morbidities (diabetes, chronic kidney disease) and laboratory parameters (leucocyte count, prothrombin time, serum ferritin, d-dimer, pro-calcitonin, fibrin degradation products, lactate dehydrogenase, neutrophil lymphocyte ratio, C-reactive protein, interleukin-6, liver function, renal function and serum electrolytes) as determinants of severe disease at the time of admission among COVID-19 patients.

Diabetes has been recognized as important in the prediction of severe disease of COVID-19. Diabetes in patients with COVID-19 was associated with a two-fold increase in mortality and severity of COVID-19, compared to non-diabetics in a meta-analysis [13]. Jain et al., studied the predictive symptoms and comorbidities for severe COVID-19 and intensive care unit admission. They concluded that elderly patients with comorbidities are more vulnerable to severe disease [14]. A systematic review by Del Sole et al included 12 studies with 2794 patients where 596 patients with severe disease. They reported patients with severe disease were older in age and had diabetes than patients with non-severe disease [15].

Del Sole identified that increased procalcitonin (OR: 8.21, 95% CI 4.48–15.07), increased D-Dimer (OR: 5.67, 95% CI 1.45–22.16) and thrombocytopenia (OR: 3.61, 95% CI 2.62–4.97) predicted severe infection [15]. A meta-analysis by Coomes and Haghbayan [16] reported that IL-6 levels are significantly elevated and associated with adverse clinical outcomes.

A study in a north Indian tertiary care centre used retrospective data to conclude that more than half of patients admitted to the hospital with SARS-CoV-2 infection had an abnormal liver function which was found to be associated with raised levels of inflammatory markers [17]. These patients had significantly higher proportions of patients with abnormal liver function were elderly and males and were at higher risk of progressing to severe disease. Organ specific manifestations, which include the liver and the kidney along with their possible mechanism of injury have been available in literature [18]. A systematic review and meta-analysis of the published studies indicate that COVID-19 incidence was higher in people receiving maintenance dialysis than in those with CKD not requiring kidney replacement therapy or those who were kidney or pancreas/kidney transplant recipients [19]. In patients with COVID-19, acute Kidney Injury (AKI) may have an inflammatory etiology mediated by a cytokine storm [20]. CKD and COVID-19 may have a higher incidence of death than people with CKD without COVID-19. [19]

Elevated levels of lactate dehydrogenase were suggested to be associated right from the early studies on COVID-19 severity. Work by Wang and Wang reported that compared to survival cases, patients who died during hospitalization had higher plasma levels of D-dimer, creatinine, creatine kinase, lactate dehydrogenase, lactate, and lower percentage of lymphocytes (LYM [%]), platelet count and albumin levels [21]. Similarly, a multicentre retrospective cohort study from Wuhan to develop and validate a prognostic nomogram for predicting in-hospital mortality of COVID-19 included age (Hazard Ratio for per year increment: 1.05), severity at admission (Hazard Ratio for per rank increment: 2.91), dyspnea (Hazard Ratio: 2.18), cardiovascular disease (Hazard Ratio: 3.25), and levels of lactate dehydrogenase (Hazard Ratio: 4.53), total bilirubin (Hazard Ratio: 2.56), blood glucose (Hazard Ratio: 2.56), and urea (Hazard Ratio: 2.14) [22].

Other parameters that we found to be associated with severe Covid-19 at admission such as leucocytosis, prothrombin time, serum ferritin, fibrin degradation products, C-reactive protein, interleukin-6, and serum electrolytes operate through a clinical pathway or indicate pathology similar to others described above.

Our study had certain limitations. There is potential for selection bias in this hospital-based study. The cases were poorer and less educated than the controls, which indicates a difference in the source population to which cases and controls belonged to. The location and type of participating hospitals could have influenced the selection of study participants. Misclassification of case-control status is unlikely as we used the standardized criteria for classification of severe cases across the study sites. There is a likelihood of misclassification of laboratory parameters, albeit minimal, on account of testing by different laboratories across the study sites. However, all laboratories were assured to have quality control mechanisms in place.

## Conclusions

We have identified a set of parameters characterizing severe Covid-19 that are part of routinely available investigations within Indian hospital settings, both public and private. Knowledge of these risk factors has the potential to triage COVID-19 patients at the time of admission in terms of severity of disease and adequate management of the same.

## Supporting information

S1 Table Definition of severe and mild disease as per Government of India’s COVID-19 case management guidelines. (DOCX)
