# Supplementary material for: Determinants of severity among hospitalised COVID-19 patients: Hospital-based case-control study, India, 2020
Source: PLoS One. 2021 Dec 29;16(12):e0261529. doi: 10.1371/journal.pone.0261529 (PMC8716035; doi:10.1371/journal.pone.0261529)
Supplement: S1 Table — (DOCX) [file pone.0261529.s001.docx]

**Supplementary material**

**Table S1:** Definition of severe and mild disease as per Government of India’s COVID-19 case management guidelines (version 5; issued on 03/07/2020)

| **Clinical Severity** | **Clinical presentation** | **Clinical parameters** |
| --- | --- | --- |
| Severe | Severe pneumonia | **Adult:** with clinical signs of Pneumonia plus one of the following; respiratory rate >30 breaths/min, severe respiratory distress, SpO2 <90% on room air.  The diagnosis is clinical; cheat imagine can exclude complications. |
|  | Acute  Respiratory  Distress  Syndrome | **Onset:** new or worsening respiratory symptoms within one week of known clinical insult.  **Chest imaging** (Chest X-ray and portable bedside lung ultrasound): bilateral opacities, not fully explained by effusions, lobar or lung collapse, or nodules.  **Origin of Pulmonary infiltrates:** respiratory failure not fully explained by cardiac failure or fluid overload. Need objective assessment (e.g., echocardiography) to exclude hydrostatic cause of infiltrates/oedema if no risk factor presents.  **Oxygenation impairment in adults:**  Mild ARDS: 200 mmHg < PaO2/FiO2 ≤ 300 mmHg (with PEEP or CPAP ≥5 cm H2O)  Moderate ARDS: 100 mmHg < PaO2/FiO2 ≤200 mmHg with PEEP ≥5 cm H2O)  Severe ARDS: PaO2/FiO2 ≤ 100 mmHg with PEEP ≥5 cm H2O)  When PaO2 is not available, SpO2/FiO2 ≤315 suggests ARDS (including in non-ventilated patients) |
|  | Sepsis | **Adults:** Acute life-threatening organ dysfunction caused by a dysregulated host response to suspected or proven infection.  Signs of organ dysfunction include altered mental status, difficult or fast breathing, low oxygen saturation, reduced urine output, fast heart rate, weak pulse, cold extremities or low blood pressure, skin mottling, or laboratory evidence of coagulopathy, thrombocytopenia, acidosis, high lactate or hyperbilirubinemia. |
|  | Septic Shock | **Adults:** persisting hypotension despite volume resuscitation,  requiring vasopressors to maintain MAP ≥65 mmHg and serum lactate level > 2 mmol/L |
| Mild | Patients with uncomplicated upper respiratory tract infection may have mild symptoms such as fever, cough, sore throat, nasal congestion, malaise, headache | Without evidence of breathlessness or Hypoxia (normal saturation). |
